# COllaborative Shared care to IMprove Psychosis Outcome (COSIMPO): study protocol for a randomized controlled trial

**DOI:** 10.1186/s13063-017-2187-x

**Published:** 2017-10-10

**Authors:** Oye Gureje, Victor Makanjuola, Lola Kola, Bidemi Yusuf, Leshawndra Price, Oluyomi Esan, Bibilola D. Oladeji, John Appiah-Poku, Benjamin Haris, Caleb Othieno, Soraya Seedat

**Affiliations:** 1 Department of Psychiatry, University of Ibadan, Ibadan, Nigeria; 2 World Health Organization Collaborating Centre, Department of Psychiatry, University of Ibadan, Ibadan, Nigeria; 3 Department of Epidemiology, Medical Statistics and Environmental Health, University of Ibadan, Ibadan, Nigeria; 4 National Institute of Mental Health, Bethesda, MD USA; 5 Kwame Nkrumah University of Science and Technology, Kumasi, Ghana; 6 University of Liberia, Monrovia, Liberia; 7 University of Nairobi, Nairobi, Kenya; 8 Stellenbosch University, Stellenbosch Central, Stellenbosch, South Africa

**Keywords:** Complementary alternative providers, Primary care, Collaboration, psychosis

## Abstract

**Background:**

Psychotic disorders are a group of severe mental disorders that cause considerable disability to sufferers and a high level of burden to families. In many low- and middle-income countries (LMIC), traditional and faith healers are the main providers of care to affected persons. Even though frequently canvassed as desirable for improved care delivery, collaboration between these complementary alternative health providers (CAPs) and conventional health providers has yet to be rigorously tested for feasibility and effectiveness on patient outcomes.

**Methods/design:**

COSIMPO is a single-blind, cluster randomized controlled trial (RCT) being conducted in Nigeria and Ghana to compare the effectiveness of a collaborative shared care (CSC) intervention program implemented by CAPs and primary health care providers (PHCPs) with care as usual (CAU) at improving the outcome of patients with psychosis. The study is designed to test the hypotheses that patients receiving CSC will have a better clinical outcome and experience fewer harmful treatment practices from the CAPs than patients receiving CAU at 6 months after study entry. An estimated sample of 296 participants will be recruited from across 51 clusters, with a cluster consisting of a primary care clinic and its neighboring CAP facilities. CSC is a manualized intervention package consisting of regular and scheduled visits of PHCPs to CAP facilities to assist with the management of trial participants. Assistance includes the administration of antipsychotic medications, management of comorbid physical condition, assisting the CAP to avoid harmful treatment practices, and engaging with CAPs, caregivers and participants in planning discharge and rehabilitation. The primary outcome, assessed at 6 months following trial entry, is improvement on the Positive and Negative Symptom Scale (PANSS). Secondary outcomes, assessed at 3 and 6 months, consist of levels of disability, experience of harmful treatment practices and of victimization, and levels of perceived stigma and of caregivers’ burden.

**Discussion:**

Information about whether collaboration between orthodox and complementary health providers is feasible and can lead to improved outcome for patients is important to formulating policies designed to formally engage the services of traditional and faith healers within the public health system.

**Trial registration:**

National Institutes of Health Clinical Trial registry, ID: NCT02895269. Registered on 30 July 2016

**Electronic supplementary material:**

The online version of this article (doi:10.1186/s13063-017-2187-x) contains supplementary material, which is available to authorized users.

## Background

Psychotic disorders are a group of severe mental disorders that cause considerable disability to sufferers and significant burden to families and caregivers. The Global Burden of Disease project estimates that, in 2010, schizophrenia was responsible for 1.1% of all Disability-adjusted Life Years due to non-communicable diseases and 2.4% of Years Lived with Disability [1, 2]. Even though relatively low-cost and effective treatments are available for psychotic disorders [3–5], only very few persons affected by these disorders receive any treatment in low- and middle-income countries (LMIC). Community studies in sub-Saharan Africa show that only about 20% of persons with severe mental disorders had received any treatment in the prior 12 months [6, 7]. A study conducted in Nigeria found that symptoms of non-affective psychoses were relatively common, with a prevalence of 1.1%. The majority of those who reported the conditions had not received any treatment [7].

It has been estimated that 80% of the world’s rural poor rely on complementary alternative health providers (CAPs) for their health care [8]. There is evidence from LMIC to indicate that CAPs, principally consisting of traditional healers (TH) and faith healers (FH)), constitute a large proportion of the de facto human mental health resource for health care service [9, 10]. Community surveys in these countries show that traditional health practitioners (THP) are often the first health care providers contacted by persons with a variety of mental health conditions [11]. There is also evidence that some patients continue with simultaneous consultations to CAPs and conventional health providers [11–13]. Studies conducted in Uganda show that about 60% of patients consulting THPs have diagnosable *Diagnostic and Statistic Manual of Mental Disorders, version IV* (DSM-IV) disorders, with about 30% of these being cases of psychosis [14, 15]. In LMIC, in particular, the prevalence and pattern of mental disorders among people attending THPs commonly reflect the beliefs within communities that people with severe mental illness are afflicted by spirits or supernatural forces for which the effective intervention is not orthodox medicine but traditional health approaches [16, 17]. Consequently, both in view of their significant presence within the health care systems in LMIC and the apparent preference for their care by many in the community, suggestions have been made for a more formal incorporation of the service of CAPs into the health system [18, 19].

Three approaches have been suggested for a more formal engagement of CAPs within the health system [11, 20]: (1) evaluation of the efficacy of traditional healing practices; (2) integration of traditional medicine into the national health system and (3) providing training for traditional healers to deliver safe and effective treatment. In view of their abundance and closeness to the community [21], there is little doubt that in most LMIC a process of collaboration with CAPs is likely to be of importance in any attempt to scale up mental health service. However, in sub-Saharan Africa, one important challenge to a formal integration of CAPs into the public mental health services is the frequent observation that CAPs sometimes use harmful practices, and abuses of human rights have been documented [22, 23]. These harmful practices include shackling of patients, beating them, and making ritual scarification, a procedure that carries a high risk of infection, including that of human immunodeficiency virus (HIV). These practices commonly reflect erroneous beliefs, which are common among both healers and the public, about the underlying causation of mental health problems and the effective interventions for them [16].

There is evidence that, with the appropriate approach, a collaborative care program can be developed between THPs and the conventional public health sector [24]. Experience, especially in the area of HIV treatment, has shown that while biomedical health practitioners may initially have reservations about collaboration [25–27], a program aimed at building mutual understanding and respect can lead to beneficial collaborations [56]. King pointed out that a key element for successful collaboration is building of mutual respect between THPs and biomedical health professionals through dialogue, exchange of information and readiness to learn from one another [24]. However, no empirical evidence has yet been provided about the possible boundaries of such collaboration in the care of persons with severe mental disorders, its feasibility, and its likely impact on patient outcome. The present study, COllaborative Shared care to IMprove Psychosis Outcome (COSIMPO) is designed to provide such evidence.

## Objectives

The main objective of COSIMPO is to compare the effectiveness of a collaborative, shared-care intervention program implemented by CAPs and primary health care providers (PHCPs) with care as usual in improving the outcome of patients with psychosis.

### Primary hypothesis

Patients presenting to CAP facilities engaged in collaborative shared care (CSC) with PHCPs will have better outcome of psychosis compared to patients in enhanced usual care at 6 months following entry into the trial as measured by a significant mean reduction in symptoms as rated by the Positive and Negative Syndrome scale (PANSS) [28]. For the purpose of this study, a 7.5-point difference on the PANSS total outcome scores between the two groups will be regarded as a clinically meaningful difference.

### Secondary hypotheses

Using outcome data collected at 3 and 6 months following trial entry, we will test the following secondary hypotheses:

1. Persons with psychotic experience treated by CAPs in the collaborative care program will be less likely to receive harmful treatment or be subject to human rights abuses (such as chaining, beating, starving, exposure to natural elements, scarification, isolation, fasting) than those in the care as usual
2. Participants in the intervention arm will be less likely to experience victimization and abuse from their caregivers. Victimization is defined as an act or action that exploits or treats a patient unfairly
3. Participants in the intervention arm will have significantly less disability in the areas of psychological, occupational and social functioning as measured with the World Health Organization’s Disability Assessment Scale (WHO-DAS) than those in the control arm

## Method/design

The study is a single-blind, cluster randomized controlled trial (RCT) being conducted in Nigeria and Ghana. The sampling frame consists of all the clusters in the 11 local government areas (LGAs) in and around the city of Ibadan in Nigeria and Ashanti region in Ghana. A cluster is composed of a primary health care clinic (PHC) and all the CAP facilities in its neighborhood, defined as the catchment area typically served by the PHC. That is, a cluster has one PHC and its neighboring CAP facilities, the number of the latter being determined by distance, ease of contact, and administrative considerations. Clusters were composed such that the CAP facilities are readily accessible to the PHCW in order to facilitate clinical engagement and support. All CAP facilities in the two study sites, previously identified and mapped during the formative stage of the project, were allocated to clusters. A total of 51 clusters were composed. Randomization of clusters was carried out before recruitment of trial participants (TPs). Allocation of clusters to each of the study arms was conducted by a statistician at the University of Ibadan (UI) Department of Epidemiology and Statistics with no other involvement in the day-to-day implementation of the study. Allocation to the two arms was balanced by site (Ghana versus Nigeria) and by size of the cluster, using the number of beds in each cluster (small versus large). Patients who are invited will not be informed of their allocation until their eligibility has been established and they have consented to participate. Fifty-one clusters were randomized into the two arms of the study across the two study sites.

### Study settings

#### Nigeria

Ibadan in Nigeria is one of the largest cities in Africa. The inhabitants are mostly of the Yoruba ethnic group. Islam and Christianity are the predominant religions, with about equal proportion of adherents, but traditional religious worshipping is also common. There are 11 LGAs in and around the city of Ibadan (five urban, six semi-urban or rural) with a current estimated total population of 3,160,200. Each of the LGAs has between 10 and 18 PHCs. All 11 LGAs are included in the study.

Specialist mental health services are provided in the department of psychiatry of a teaching hospital, a psychiatric unit of a general hospital, and one private hospital, by a combined total of 12 psychiatrists. These orthodox facilities have a total of about 120 psychiatric beds. There are about 71 facilities run by complementary alternative health providers, made up of traditional and faith healers, and providing mental health care. These facilities have an estimated total of about 430 admission spaces at the time of the study.

#### Ghana

The Ashanti Region is located in south Ghana. It is the third largest of 10 administrative regions in the country but the most populous, with an estimated 2010 population of 4,780,380, accounting for 19.1% of the country’s population. Specialist mental health services are provided by two psychiatrists working in a department of psychiatry of a general hospital with 39 admission beds. The region has more than 120 CAPs providing mental health care in facilities with a total of 430 admission spaces. Seven districts were selected for the study in Ashanti on the basis of having the size of population required for the study and being accessible to the research center.

### Inclusion criteria

Potential trial participants (TPs) must be:

1. Aged 18 year or over and speak the study language of Yoruba (Nigeria) or Twi (Ghana)
2. An inpatient at participating CAP facilities in the selected clusters with a confirmed diagnosis of non-organic psychosis as assessed by research interviewers using the Structured Clinical Interview for Diagnostic and Statistical Manual version IV (SCID) [29]
3. Symptomatic at the time of recruitment as indicated by a minimum score of 60 on the total Positive and Negative Symptoms Scale (PANSS) scale [28]

### Exclusion criteria

1. Patients with serious physical illness and in need of urgent medical attention (e.g., serious infection, injury, etc.) as determined by the research assistants (RAs), a PHCP or by the independent social worker during the process of assessing for capacity to consent. This may be evident from the presence of high-grade fever (temperature of 40 °C or greater), apparent injury, state of consciousness or evidence of profound confusion
2. Serious cognitive impairment that may interfere with research assessment, such as having a prior history of intellectual or learning disability as assessed clinically by an independent social worker
3. Persons who would not be in the study area for at least 6 months following recruitment, such as patients brought in from other parts of the country outside the study area who may return to their usual domicile far from the study area after the period of treatment. This information is obtained from caregivers by the RAs
4. Women who are pregnant or attempting to become pregnant during the study period. Pregnancy testing will be conducted on all eligible female potential trial patients by a research staff that has been appropriately trained. Research assistants will seek the assistance of the CAP to obtain a urine sample from the potential female TP. Once a sample is obtained, the RA will use a pregnancy test strip to test for pregnancy. Only those whose test results suggest that they are not pregnant will be enrolled into the trial. Eligible female participants who decline to have pregnancy test will be excluded. Pregnant women are excluded because special considerations and precautions might be required in the decision to prescribe antipsychotic medications for them that would fall outside the procedures within the collaborative shared care. Such considerations might require the expertise and determination of a physician. Physicians are not involved in the routine care of patients recruited to COSIMPO except through referral from PHCPs

#### Informed consent procedure

Participants are recruited into the study either following contact of the research team by the treating CAP or during regular routine visits to the CAP facilities by the RAs. All consecutively admitted patients considered by the participating CAPs to have psychotic disorders at the time of recruitment are approached for possible inclusion in the trial. Two levels of consent are sought from patients: consent to be screened for eligibility, and, if eligible, consent to participate in the trial (see section on “Declaration” below).

#### Schedule of trial recruitment and participation

**Fig. 1:**
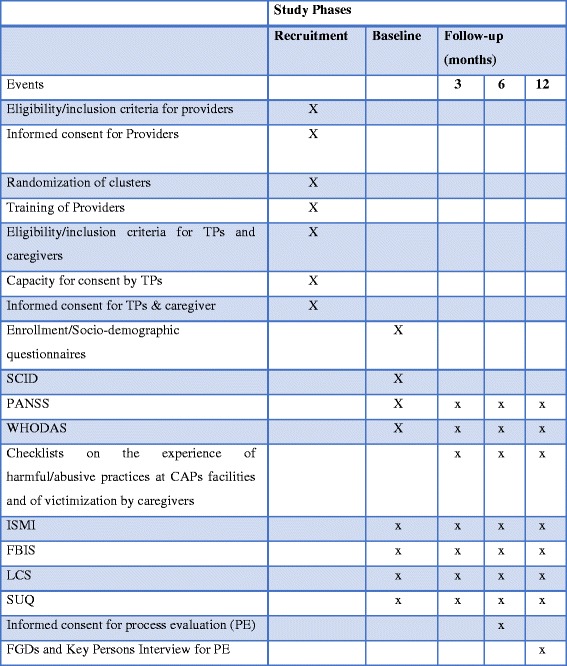
Schedule of enrollment, intervention and assessments

Enrollment into the study commenced on 19 September 2016 and is projected to end 30 June 2017. The last 6-month follow-up assessment is expected to occur in December 2017. The schedule of enrollment and assessments is as in the Standard Protocol Items: Recommendations for Interventional Trials (SPIRIT) Figure (Fig. 1).

### Training of providers (see Table 1)

**Table 1:** Schedule of training

	Description of training
Primary care providers in the intervention arm	3-day interactive training consisting of (1) a review of the clinical features of psychosis, including the assessment, diagnosis and evidence-based management of psychosis, using the mhGAP Intervention Guide; (2) the roles of the different levels of the mental health system in the care of persons with psychosis; (3) the structure and components of the collaborative shared care; and (4) expectations, roles, barriers and facilitators for effective collaboration
Traditional and faith healers in the intervention arm	2-day interactive training consisting of (1) discussion about their understanding of the concept of psychosis and information about the medical nature of psychosis, including symptoms and course of illness; (2) a review of the conventional and complimentary approaches to the care of persons with psychosis; (3) identification of potentially harmful treatment practices and possible ways to avoid their use; (4) basic concepts of human rights; (5) the structure and components of the collaborative shared care; and (6) expectations, roles, barriers and facilitators for effective collaboration
Primary care providers in the control arm	2-day interactive training consisting of (1) a review of the clinical features of psychosis, including the assessment, diagnosis and evidence-based management of psychosis, using the mhGAP Intervention Guide and (2) the roles of the different levels of the mental health system in the care of persons with psychosis
Traditional and faith healers in the control arm	1-day interactive training consisting of (1) discussion about their understanding of the concept of psychosis and the information about the medical nature of psychosis, including symptoms and course of illness; (2) a review of the conventional and complimentary approaches to the care of persons with psychosis; (3) identification of potentially harmful treatment practices and possible ways to avoid their use; and (4) basic concepts of human rights

Participating providers in both arms of the study received structured and targeted training prior to trial commencement. Training was conducted separately for CAPs and PHCPs. This separation was necessary to ensure the use of appropriate presentational approaches, including language and content of training, which was designed to be appropriate for the level of education and literacy of these two distinct groups of providers. For example, the training of the PHCPs, and not that of the CAPs, included the use of the WHO Mental Health Gap Action Program Intervention Guide (mhGAP-IG) [30] in the management of psychosis. The local language of the participating sites was used during the training of the CAPs at each of the sites. Role-plays on specific components and contents of the collaborative care were modelled at these sessions. An important component of the training of the PHCPs in the CSC arm was on dialogue and engagement with CAPs. While formal and written pre- and post-training tests were conducted for the PHCPs, the CAPs were assessed verbally by asking participants to describe individually the components of the collaborations as well as steps to take when presented with different clinical scenarios. The training of the PHCPs was based on specific defining features of the collaboration as well as on recognition and treatment of psychosis, including the identification and response to adverse events and medication side effects. Treatment approaches for psychosis followed the mhGAP-IG specifications, adapted, where necessary, for the context of their use in a CAP facility. Training of both groups included an understanding of the range of possibly harmful and abusive practices and how to avoid them.

### Interventions in COSIMPO

#### Intervention arm

The collaboration between the CAPs and the PHPCs taps into the skills of both types of providers for the management of patients with psychosis. The patients who are treated within this collaborative program have access to both orthodox medical care as well as complementary alternative medical approaches. However, it is important to note that, in the design of COSIMPO, patients belong to the CAPs primarily and the PHCP acts only in advisory and supportive capacity in patient management. The role of the PHCP is to help the CAP to provide safe and acceptable care to patients, including promotion of, and respect for, human rights and the avoidance of harmful practices. The collaboration is based on mutual respect, trust, support, referral and advice between the CAPs and PHCPs. In the collaborative arrangement, psychotropic medications are to be prescribed only by trained orthodox health care personnel, which is the PHCPs in the first instance or physicians or specialists if referrals are made to them by the PHCP following consultation with the CAP. CAPs in the intervention arm commit themselves to implementing enhanced patient care through the collaborative arrangement with PHCPs.

In providing clinical support to the CAP, the PHCP engages with the CAP, the patient, and the caregivers of the patient. The PHCP conducts regular, weekly, scheduled personal contacts with the CAPs as part of the collaboration, using such contacts to reinforce information on best clinical practice, provide information on patient rehabilitation, and attend to other clinical issues that the CAPs may bring to the attention of the PHCP. Beyond the regular visits, the PHCP also responds to requests from the CAP for urgent clinical needs as determined by the CAP. Such needs may include acute deterioration of TPs’ mental status, including instances of threats of violence or absconding, or emergent or worsening physical illness.

There are two main components of the collaborative shared care:

1. Clinical support to respond to the medical need of psychotic patients: this involves the provision of medical care to patients, especially those in conditions of acute psychotic disturbance, by the PHCP following the request from CAP. This will often entail the administration of psychotropic, especially antipsychotic, medication
2. Clinical support to improve service on a continuous basis: this involves engagement and interactions between PHCP and CAP during which the former provides the latter with specific information to improve service, organize rehabilitation for their patients, and generally provide better care. This interaction will involve the provision of specific information by the PHCP to the CAP during scheduled and regular weekly visits

All interactions and activities are documented fully by the PHCP in each TP’s clinical records. These two activities will form the basis of the evaluation of the collaborative care both in regard its feasibility, the fidelity of its implementation, as well as its impact or effectiveness on patient outcome.

When a PHCP is involved in the care of a TP, the PHCP assesses the patient at the CAP facility, provides feedback to the CAP, and initiates treatment according to treatment specifications for psychosis as described in the mhGAP-IG. The PHCP continues to monitor the patient on at least weekly regular visits, but a more frequent monitoring may be required, depending on the clinical need of the patient (for example, administration of specific medications may require daily or more visits to monitor their effects). If the patient is taking herbal medication, the PHCP takes this into consideration in administering psychotropic medications. The PHCP may refer a TP to see a physician or specialist, as necessary, but always in consultation with the CAP.

#### Control arm

Providers in the control arm offer enhanced care as usual (CAU). Usual care means that provides will operate as they currently do, without a structured approach to collaboration between CAPs and PHCPs. However, the care provided by the healers is enhanced through interactive training workshops (see Table 1). For the CAPs, the training consisted of (1) discussion about their understanding of the concept of psychosis and the information about the medical nature of psychosis, including symptoms and course of illness; (2) a review of the conventional and complimentary approaches to the care of persons with psychosis; (3) identification of potentially harmful treatment practices and possible ways to avoid their use; and (4) basic concepts of human rights. For the PHCPs, the training consisted of (1) a review of the diagnosis of psychosis; (2) evidence-based management of psychosis, using the mhGAP-IG; and (3) the roles of the different levels of the mental health system in the care of persons with psychosis. While the training of the CAPs was over 2 days, that of the PHCPs was over 1 day. The goal of the trainings was to improve the care of persons with psychosis by both groups of providers in the context of current practice in which formal collaboration does not occur.

### Responses to harmful treatment practices

COSIMPO is designed to engender collaboration between CAPs and PHCPs to enhance the outcome of patients with psychosis. CAPs, in the course of delivering care to persons with severe mental health disorders, including psychotic disorders, may use what can be regarded as potentially harmful or abusive approaches. These approaches are largely informed by their understanding of the nature of psychosis by CAPs and by the need to curtail difficult clinical situations. For example, CAPs might believe that patients who are aggressive are possessed of evil spirits and that physically beating such patients might drive out the spirits. Also, in handling patients who are violent and at risk of harm to self or to others, CAPs might feel the need to shackle such patients to restrict their movements and the attendant risk of violence. All of these possible scenarios that might lead to the use of harmful or abusive practices are addressed within COSIMPO through training of the providers, as described in this protocol, and through the implementation of the collaboration with PHCPs who are able to administer medications to calm agitated and potentially violent patients.

There is need to reduce risks to all the TPs (in both intervention and control arms) as much as possible while getting the scientific and socially valuable information the study has potential to offer. To this end, a schedule of responses to harmful practices is developed to guide the research team to ensure that the team is not complicit to the use of harmful practices by CAPs while at the same time keeping the CAPs sufficiently engaged in the study in order to answer the study questions. The schedule sets out a typology of responses to some of the more common forms of harmful practices that may occur in either arm of the study. Responses are determined by the severity of the practice (see Table 2). CAPs who engage in harmful practices are expected to cooperate with the research team in the execution of the responses to these practices and to reduce such practices.

**Table 2:** Schedule of response to harmful practices

Practice observed	Thresholds of harm	Responses control arm	Responses intervention arm
Shackling	1. Short-term (hours) 2. Infections due to long-term shackling	1. Continued monitoring 2. Study team works with CAP to treat infections (by facilitating access to medical care)	1. PHCP consultation and study team consultation to employ alternative measures 2. PHCP will treat the infection; if more medical or specialist care is required, PHCP will initiate referral to such care
Beating	1. Evidence of beating – equipment for beating present at site 2. Evidence of beating particular patient (marks, scars)	1. Continued monitoring 2. Continued monitoring	1. PHCP consultation and study team consultation not to beat patients 2. PHCP to reinforce the need to avoid practice
Scarification	1. Wounds have healed 2. Wounds are yet to heal	1. Continued monitoring 2. Study team works with CAP to treat infections(by facilitating referral to hospital)	1. PHCP to reinforce the need to avoid practice 2. PHCP will treat infection and reinforce the need to avoid practice (and if necessary, initiate referral to medical care)
Herbs	1. Patient shows non- serious signs of side effects or drug reaction (e.g., vomiting, skin rash, etc.) 2. Patient is seriously unwell as a reaction to herbs	1. Continued monitoring 2. Work with CAP to treat illness (by facilitating referral to hospital)	1. PHCP monitors the situation and offers suggestions about avoiding herbs 2. PHCP will assess and treat the condition; if more medical or specialist care is required, PHCP and study team will facilitate access to such care
Sexual abuse	1. Any report or other evidence of sexual abuse	1. Study team reports to legal authorities	1. Study team reports to legal authorities

*CAP* complementary alternative health provider*,PHCP* primary health care provider

Disengagement of a CAP from the study will occur at a defined instance of continued refusal to cooperate with the study team in implementing these harm-reduction responses. Given that harmful practices are often a part of the standard of care of some CAPs and are among what the project sets out to change, direct intervention by the research team will be determined by the need to ensure that the patient’s safety is promoted while ensuring that the scientific objectives of the study are accomplished. The decision about when and how the research team will take action or intervene with patients and/or caregivers to ensure the protection of the patients against the consequences of harmful practices is be guided by specific rules. These rules are set out to enhance patient safety while giving sufficient scope for the trial to meet its scientific objectives.

Thus, the PHCP will respond to harmful practices in the intervention arm by providing appropriate treatment to the patient and facilitating hospital referral when necessary. PHCPs will also directly intervene with the patients and their caregivers by providing advice on seeking alternative treatment in instances of CAPs refusing to cooperate in the management of a case of life threatening or “severe” harmful practice or when such practice has been observed on more than two occasions at a particular CAP facility. In the control arm, where PHCPs are not involved, the trial team will perform the role of engagement with patients and caregivers using the decision rules described in Table 2.

Information on observed harmful practices will be documented by the RAs and the PHCPs in a form designed for the purpose to track the type of harmful practice, the rating of its severity, the immediate response of the PHCP or RA to the observed practice, the outcome and planned follow-up action.

### Outcome evaluation

Outcome measures will be undertaken at 3 and 6 months by RAs.

*Primary outcome*, to be assessed at 6 months, will be the mean difference in recovery rate from psychosis as measured by improvement or reduction in mean PANSS total scores of patients in intervention arm (from baseline) compared to those in the CAU arm. The PANSS is chosen for its demonstrated acceptability and validity, its proven sensitivity to change and our experience with its use in our previous and on-going studies in Nigeria.

*Secondary outcomes*, to be assessed at 3 and 6 months, include levels of disability, of self-stigma, the experience of harmful treatment practices and of victimization by caregiver as well as the course of the psychotic illness in four key domains (symptoms, treatment, residence and work.

### Assessment instruments

The psychosis section of the Structured Clinical Interview for DSM-IV Axis 1 disorders (SCID) is used to confirm the diagnosis of psychosis. The SCID is a semi-structured interview for making the major DSM-IV Axis 1 diagnosis. Information collected by trained RAs using the SCID is reviewed by research psychiatrists to assign subtype diagnosis of psychosis and make a rating on course of the psychosis illness. The Positive and Negative Syndrome Scale (PANSS) [28] is used to measure symptom severity of psychosis at baseline, 3 and 6 months. Disability is being measured with the World Health Organization’s Disability Assessment Schedule 2.0, a generic tool for assessing health and disability [31]. The Proxy version of WHO-DAS is used when a TP is unable to provide reliable information. The 29-item Internalized Stigma of Mental Illness (ISMI) scale [32] is used to assess level of self-stigma. The burden of caring for TPs by their caregivers is being assessed with the Family Burden Interview Schedule (FBIS) [33], a standardized instrument designed for the. The Life Chart Schedule (LCS) is being used to assess the long-term course of the psychotic illness in four key domains (symptoms, treatment, residence and work) [34]. To capture the range of services and the cost associate with their use, we use the Service Utilization Questionnaire [35] (adapted for COSIMPO). The SUQ systematically collects resource-use data, including cost of care received from CAPs, consultations with PHCPs and with other conventional health providers, use of herbs and drugs, cost of treatment, including those of rituals and sacrifices, and any other service use-related costs. Checklists of harmful treatment practices, human rights’ abuses and of victimization, developed with information obtained during the formative stages of the Partnership for Mental Health Development in sub-Saharan Africa (PaM-D), are used for the indicated purposes. These assessments are conducted through both observation during visits by the RAs to CAPs facilities and by patient interviews. For example, respondents are asked if they have experienced any of the practices or abuses in a defined period of time (at the baseline, this will be in the previous 3 months, and at follow-up assessments, this will be “since the last time we spoke with you”). Every positive answer is followed by exploration of the number of times that the event had been experienced or the duration of its occurrence, whichever is relevant to the event.

In order to assess the extent to which collaboration affects the knowledge and attitude of the CAPs, we administer the Knowledge About Schizophrenia Interview (KASI) [36], a semi-structured interview that enquires about respondent’s view about the diagnosis, symptomatology, etiology, course, prognosis and management of schizophrenia, to the CAPs participating in the study at the baseline and at the 6-month primary endpoint.

### Discontinuation criteria

Trial participants will be discontinued from the trial at any point in the following instances:

1. Development of a life-threatening physical illness in which a referral to hospital is strongly advised
2. Serious suicidal intent (indicated by a suicidal plan or attempt)
3. Becoming pregnant (confirmed by a detailed history of menstruation and a urine test)
4. Incarceration

Patients, who during the routine weekly visit by the research team or by the PHCP, are reported or observed to meet any of these criteria, will be assessed immediately by the trial manager on whose recommendation the site leader will decide on possible discontinuation. The data collected on any patient who discontinues from the study will be retained in the study up to the time of the discontinuation and included in the analysis on an intent-to-treat basis.

### Blinding

TPs are not blinded to their treatment arms (as it is impossible to conceal the fact of collaboration or lack of it from the patients). There are two groups of trained assessors: the RAs who conduct the baseline and 3-month assessments, and those conducting the 6-month primary outcome evaluations. While the former cannot be blind to patient trial arm (since they will conduct the baseline assessment at the recruitment facility and the TP may still be on admission at that facility for the 3-month outcome assessment), the latter will be kept blind to trial allocations. TPs will be instructed not to disclose whether or not they are receiving the intervention to the primary outcome assessors. The primary outcome measure (PANSS) will always be completed first in order to minimize the risk of bias in the event of unmasking during the assessments and, if it occurs, the point of unmasking will be recorded. Sensitivity analyses will be carried out to assess the effect of unmasking on the primary outcomes.

### Data collection and quality control

Data collection and capture are regulated by a series of specific steps described in the Data Management Protocol. The protocol specifies steps to be taken to ensure data integrity and quality.

Data is being collected using pen and paper. Collected data is checked for accuracy and consistency by the RA, the supervisor, the trial manager and the data-entry clerk before it is entered using the Mobenzi software. Mobenzi obeys series of programmed instructions such as “Skip patterns” and thus ensures seamless data entry and quality. At the end of every entered interview, Mobenzi prompts a message pop-up to request the acceptance or rejection of the case.

Field work is monitored by research supervisors to ensure that assessments are conducted according to protocol specifications. Supervisors make random checks on the quality of interviews (by physically observing at least 10% of all interviews conducted by an RA) and randomly check the correct entry of study data by RAs. The supervisors work under the direction of the trial manager. The supervisors provide first-line response to research and clinical issues as they arise in the field and consult or refer difficult issues to the site trial manager. Any unanticipated problem encountered by an RA is reported to the supervisor who is required to either solve the problem or report the problem to the trial manager. Problems that cannot be solved by the trial manager are referred to the site leader or the principal investigator (PI).

The trial managers at both sites are responsible for the coordination of the field work and are responsible to the Trial Management Team through the site leader (in Ghana) or the PI (in Ibadan). The trial manager at each site ensures protocol adherence and monitors the work of the RAs and research supervisors.

### Data protection process

In order to ensure safety of data, collected data is entered into password-protected computers by data-entry clerks. Data will be backed up on another computer (also password-protected and assessable only to the data manager. Data captured in this way is, thereafter, transferred to the server/domain through the File Transfer Protocol (FTP) synchronization process of Mobenzi. The data manager at each site is responsible for uploading field data via the Internet to an off-site server. The data manager alone will have the access to the domain through a user name and password for data protection and security purpose.

All paper data files (quantitative and qualitative) are being stored in lock-and-key cabinets and the computerized data will be password-protected. Any back-ups made of the computerized data are put on separate hard drives which will also be password-protected. Only named research staff have access to the data onsite.

### Data monitoring

The trial is monitored by an independent Global Data Monitoring and Safety Board (DSMB) constituted by the National Institute of Mental Health (NIMH), the trial funders, as well as local, independent Institutional Review Boards (IRBs) at the UI and the Kwame Nkrumah University of Science and Technology, Kumasi.

**Fig. 2:**
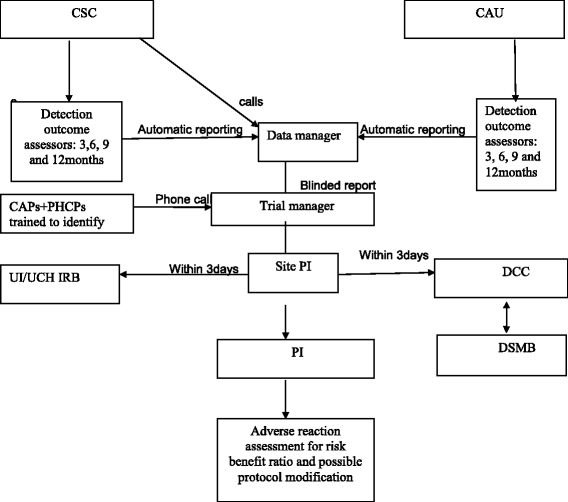
Reporting diagram of serious adverse events (SAEs)

The NIMH Global DSMB will ensure the safety of TPs. The board, as well as the local IRBs at the study sites, have reviewed and approved the study protocol, informed consent and all relevant documents. The other main responsibilities of the DSMB and the IRBs are, but not limited to, the following:

- □ Review of protocols, consent procedures, Consent Forms and safety plans prior to the initiation of the study
- □ Monitoring of the progress of the study, including recruitment and retention of participants, adverse events, serious adverse events (SAEs) (which will be reported as shown in Fig. 2), reasons for participant withdrawal, adherence to the timeline of the study, quality of data, and protocol violations
- □ Make definitive and authoritative decisions about the continuation, modification or termination of the study based on the balance of adverse events and beneficial outcomes. The data and safety monitoring plan will involve the continuous evaluation of safety, data quality and data timeliness. The PI will be expected to continuously review data and patient safety which will be reported at Trial Management Committee (TMC) and Trial Steering Committee (TSC) meetings and all discussions will be documented. The PI will also submit twice yearly progress report to the NIMH DSMB.

An independent professional trial monitoring organization is engaged by the funders to conduct random and scheduled auditing of the trial. The monitors conducted site initiation to flag off patient recruitment and have conducted three other visits to both study sites at the time of this report. Site monitoring reports are provided to the DSMB as well as to the study Institutional Review Boards.

### Sample size and potential power of the trial

The primary outcome is symptom severity as assessed with the PANSS score. Based on our previous naturalistic follow-up study of patients with psychosis undergoing treatment [37], we estimate that a mean difference of 7.5 units on the total PANSS outcome scores between the two arms will represent a clinically significant difference (given that a standard deviation of 20 was obtained for the PANSS in that study). With the target effect size of 0.38, an uninflated sample size of 112 patients per arm will be required to provide a power of 80% and at an alpha of 0.05. We expect to be able to recruit an average total of six patients per cluster. To take account of the cluster design, we inflate the estimated sample size by 1 + ((*k* − 1) × *ICC*)) where *k* is cluster size for analysis and *ICC* is the intra-cluster correlation coefficient. We estimate an *ICC* of 0.02 for the PANSS in primary care settings. Using the resulting design effect of 1.10, the inflated sample size is 246.4 (224 × 1.10). With an estimated attrition of 20% at 6 months (an estimate supported by our experience with the follow-up efforts so far) we will need to recruit a total of 296 participants. A total of 49 clusters (rounded up to 50) will be required to reach the target number of participants. We have total of 51 clusters across the two study sites and all are included in the trial.

### Data analyses

**Fig. 3:**
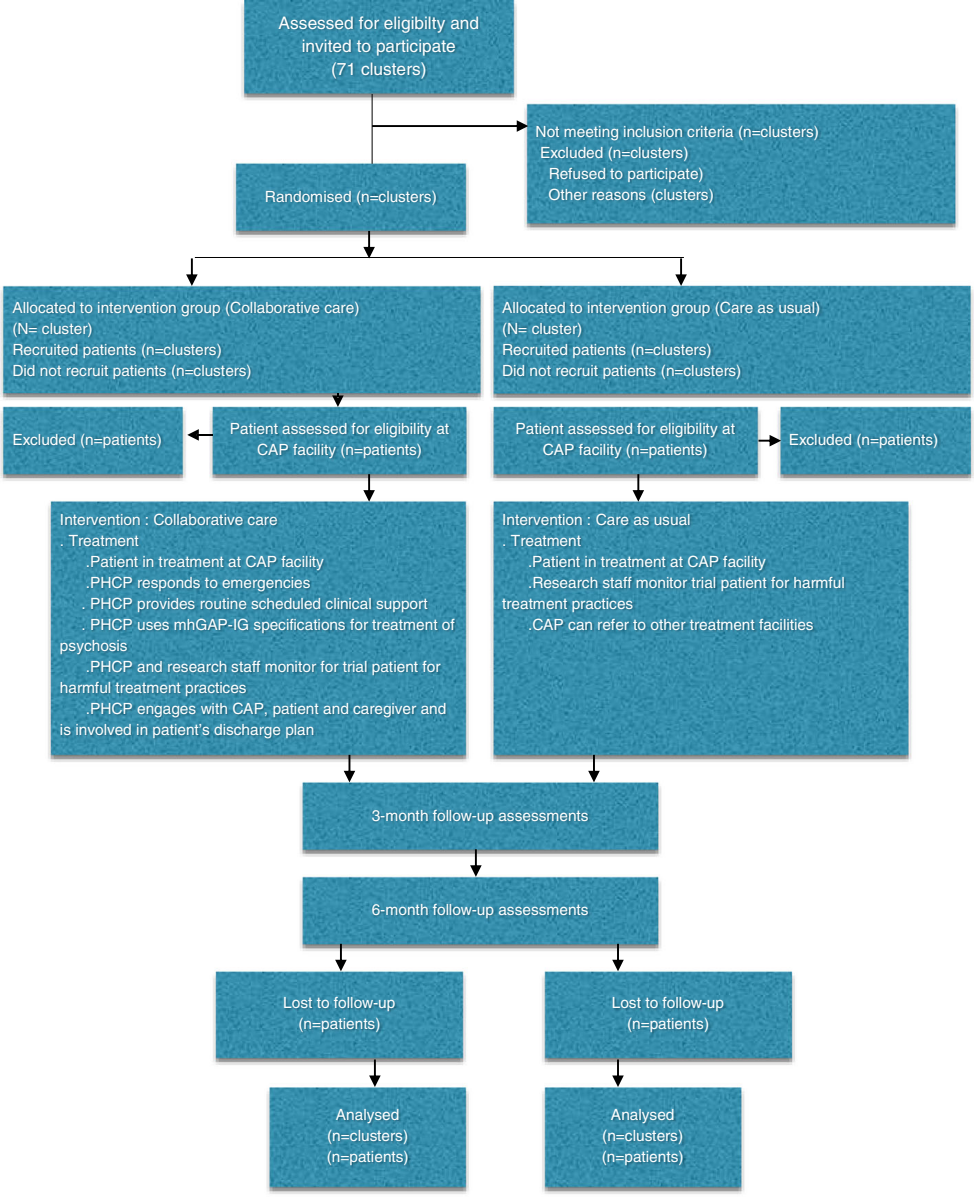
COllaborative Shared care to IMprove Psychosis Outcome (COSIMPO) trial flow chart

Initial analyses will include comparisons of baseline characteristics of individuals who consented and did not consent, and participants who could and could not complete baseline assessments, and of the distribution of potential confounding factors. Findings will be reported as per Consolidated Standards of Reporting Trials (CONSORT) guidelines for cluster randomized controlled trials [38] including a trial flow chart (see Fig. 3). This will include the flow of clusters and participants through each stage of the trial, including the number eligible, randomly assigned, receiving the intended treatment, completing the study protocol and analyzed for the primary outcome.

The outcome measures will be summarized at baseline, 3-month and 6-month follow-ups by intervention arm and overall. These will be summarized by means (SD), medians (IQR) or numbers and proportions as appropriate (and including age, gender, baseline outcome score), adjusting for cluster. For continuous outcomes, histograms within each arm will be plotted in order to assess how closely the scales follow a normal distribution to determine how to describe the outcomes and how to properly do the inferential analysis. The clusters will be described in terms of gender, education, country site, and profile of the CAP.

### Outcome analyses

The primary analyses will be intention-to-treat at the 6-month follow-up visit adjusted for baseline measures. That is, after randomization, patients will be analyzed according to their allocated treatment irrespective of the treatment that they actually received. These analyses will also disregard adherence to the intervention or withdrawal from the trial. Random-effects logistic regression will be used to analyze binary outcomes, adjusting for country site and cluster size as random-effects variables. Analyses of continuous outcomes will use random-effects linear regression, additionally adjusting for the baseline value of that outcome. Adjustments will also be made for any a-priori-defined sets of potential confounding variables for which randomization did not achieve balance between the two arms at baseline. This may include, for example, differences in the type of CAPs (e.g., traditional healers versus faith healers). Effect sizes will be reported as: (1) crude and adjusted relative risks (ARRs) estimated using the marginal standardization technique with 95% confidence intervals for the ratios estimated via the delta method [46] for binary outcomes and (2) as mean differences and standardized mean differences (SMDs), with 95% confidence intervals for continuous outcomes. Missing data on the outcomes will be estimated using multiple imputations in Stata. Secondary analyses will include repeated measures analyses of data collected at 3 and 6 months, which make efficient use of all available data. No interim analysis of outcomes is planned.

### Economic measures

An economic evaluation is included in COSIMPO. For this purpose, we will use the Service Utilization Questionnaire (SUQ), adapted for the purpose, to systematically collect resource-use data, including any inpatient care, consultations with health providers, use of drugs and laboratory tests, as well as payment in kind (such as chickens and goats) and the offering of sacrifices and rituals For this exercise, we will use previously computed estimates of unit costs for resource inputs related to the use of public facilities, including primary health care. We expect an increase in the use of conventional services by the patients in the intervention arm. On the other hand, patients in the control arm may engage in more financially burdensome visits to providers who offer less effective care. Since psychosis and associated disability outcomes for the collaborative care intervention are expected to improve significantly, the intervention will “dominate” usual care (i.e., better outcomes, less cost). Such a hypothesis negates the need for a power calculation. If, however, costs turn out to be higher in the intervention group, bootstrapped incremental cost-effectiveness ratios for PANSS and WHO-DAS disability scores level will be derived. 

### Dissemination and data-sharing policy

A publication policy has been designed and approved by the investigators to guide authorship of publications. Other than dissemination through academic channels (journals and conferences), dissemination workshops are planned for CAPs and PHCPs at the study sites at the end of trial and analysis of results.

Our data-sharing procedure will be guided by the following principles: (1) the need to ensure that the datasets are first used to address the primary aims of the project (which will be done within 2 years of data collection); (2) every effort will be made to offer unrestricted access thereafter, with the only proviso being the continued protection of the anonymity of participants; and (3) due acknowledgment is given by subsequent users to the original source of the data of the funding. These conditions are in line with the NIMH data-management and data-sharing policy. We will work to ensure that the data, whenever it becomes available to the public, is in the form that can be understood and used by the research community.

## Discussion

There is a large treatment gap for people with psychosis and other mental disorders that is far more evident in LMIC where there is a dearth of mental health professionals [6, 39]. Bridging this treatment gap in the foreseeable future will involve the use of non-specialists whose training takes fewer years than those of specialists and who are, therefore, more likely to be available in adequate numbers to bridge the existing treatment gap in LMIC [40]. One common approach to utilizing these available human resources is the use of task-shifting, whereby primary health care workers are trained and supervised to take on more roles in the care of persons with mental health conditions [41]. This task-shifting or task-sharing approach has been deployed to varying degrees of effectiveness in attempts to scale up services for different conditions [41].

Other than inadequate numbers of specialists to meet the need of persons in need of mental health service, there is also the factor of cultural belief that determines where patients with psychotic disorders and their caregivers seek care [11]. Because of the shared understanding of the nature of psychosis by the lay public and providers of complementary health care, patients with psychotic disorders are commonly drawn to the care of traditional and faith healers who are also much more easily accessible than mental health specialists. Given this reality, a need to find a way of incorporating the services of the healers into the mainstream public health system has often been canvassed [18, 19]. However, in order to do this, empirical evidence for the feasibility and effectiveness of a formal engagement of the healers with the public health system is required. Such evidence will, among other goals, also assess to what extent is it possible to improve the care provided by the healers especially in regard to a reduction in the use of harmful and abusive treatment practices.

COSIMPO is designed as a test of the feasibility of task-shifting for the care of persons with psychosis not only to primary care providers, through training in the delivery of evidence-based care for such persons, but also through collaborative shared care for psychosis delivered by these providers and complementary health providers. The study is designed to provide empirical evidence for the effectiveness of this approach in responding to the treatment gap for severe mental disorders. The promise of the trial is not only in regard to providing such evidence, but in also determining to what extent a collaborative shared care between traditional and faith healers, on the one hand, and primary health care workers, on the other hand, can lead to a reduction in the use of potentially harmful treatment practices by the former. Addressing the concern about the use of such practices in the treatment of severe mental disorders will be an important contribution to the discussion about the feasibility of integrating these healers into the mainstream mental health services in LMIC.

The design of COSIMPO takes account of several important features that, from our formative studies, are crucial in engendering trust and mutual respect. The entry point to treatment for patients will be from the traditional healers or faith healers (i.e., CAPs). The CAPs take responsibility for the patient and commence treatment. The contract to care for the patient therefore lies with the CAP. The discretion about when to seek clinical support from or refer to the PHCP also lies with the CAP. The PHCP in turn provides the necessary support and assistance needed. In providing clinical support to the CAP, the PHCP may engage with the CAP, the patient, and caregivers of the patient. Beyond specific referrals and consultations, the PHCP will maintain regular weekly scheduled personal contacts with the CAPs as part of the collaboration, using such contacts to reinforce information on best clinical practice, provide information on patient rehabilitation, and attend to other clinical issues that the CAPs may bring to the attention of the PHCP.

The results of our formative work with CAPs showed that their treatment modalities can be broadly grouped into (1) harmful, (2) innocuous and (3) possibly helpful. Harmful practices include shackling, beating/physical assaults, scarification, imposed long periods of fasting (starvation) and isolation. PHCPs will discourage these practices and offer to assist in managing patients who are being exposed to such practices, possibly as a result of the severity or challenging nature of their illness. During on-going engagement with the CAPs, PHCPs will always be mindful of the need for sensitivity, respect and avoiding undermining the authority of the CAPs. Innocuous practices are not harmful to the patients and very often reflect deep cultural and religious beliefs shared by both patients/relatives and CAPs. Examples include the use of sacrifices and rituals. PHCPs will be trained and encouraged to remain open-minded about the use of innocuous practices and to leave deeply held belief systems undisturbed provided that they do not have the potential to result in harm. Possibly helpful practices within the CAM system include the use of herbs, animal products and minerals. For the purpose of potential referral/consultations to the PHCP, it is important that PHCPs are aware of the content and side effects of herbs, so as to be able to manage them if the need arises. For this reason, CAPs who use herbs will be encouraged to provide the names of the herbs. Research team will seek to obtain the active ingredients of such herbs from the literature. PHCPs will be provided with this information as well as ways to respond to any adverse events in the patients who are administered such herbs and take appropriate action.

Cluster randomized controlled trials have peculiar challenges. One of such challenges is that of possible contamination. This was minimized in this study by stratifying eligible and consenting clusters by LGA before allocation to the CSC or the CAU arm using a computer-generated random number sequence, thus avoiding contiguous clusters being in the same arm. Furthermore, the availability of collaboration in the CSC arm was not publicized to other CAPs. The stratification of the clusters also took account of size, thus limiting the potential limitation of differential recruitment into the arms of the study. We have continued to monitor the recruitment of participants into the arms. Subsample selection bias has been avoided in this study by recruiting all eligible and consenting participants from randomized clusters, thus maintaining the power of the study.

### Trial status

The protocol version number is 14.0 submitted on the 7th December, 2016. Enrollment into the study commenced on 1st September, 2016 and projected to end June 30, 2017.

## Additional file

Additional file 1: Showing the SPIRIT Checklist for the COSIMPO trial. (DOC 123 kb)

## Abbreviations

ARR: Adjusted relative risk
CAP: Complementary alternative health provider
CAU: Care as usual
CONSORT: Consolidated Standards of Reporting Trials
COSIMPO: COllaborative Shared care to IMprove Psychosis Outcome
CSC: Collaborative shared care
DCC: Data Coordinating Centre
DSMB: Data Safety and Monitoring Board
DSM-IV: *Diagnostic and Statistical Manual of Mental Disorders, version IV*
FBIS: Family Burden Interview Schedule
FH: Faith healer
FTP: File Transfer Protocol
HIV: Human immunodeficiency virus
ICC: Intra-cluster correlation coefficient
IQR: Interquartile range
IRB: Institutional Review Board
ISMI: Internalized Stigma of Mental Illness
KASI: Knowledge About Schizophrenia Interview
LCS: Life Chart Schedule
LGA: Local Government Area
LMIC: Low- and middle-income country
mhGaP-IG: Mental Health Gap Action Programme-Intervention Guide
NIMH: National Institute of Mental Health
PaM-D: Partnership for Mental Health Development in sub-Saharan Africa
PANSS: Positive and Negative Syndrome Scale
PHC: Primary health centre
PHCP: Primary health care provider
PI: Principal investigator
RA: Research assistant
RCT: Randomized controlled trial
SAE: Serious adverse event
SCID: Structured Clinical Interview for DSM-IV
SD: Standard deviation
SMD: Standard mean difference
SPIRIT: Standard Protocol Items: Recommendations for Intervention Trials
SUQ: Service utilization Questionnaire
TH: Traditional healer
THP: Traditional healing practitioner
TMC: Trial Management Committee
TP: Trial participant
TSC: Trial Steering Committee
UCH: University College Hospital
UI: University of Ibadan
WHO: World Health Organization
WHO-DAS: World Health Organization Disability Assessment Schedule
